# Genome wide signatures of positive selection: The comparison of independent samples and the identification of regions associated to traits

**DOI:** 10.1186/1471-2164-10-178

**Published:** 2009-04-24

**Authors:** William Barendse, Blair E Harrison, Rowan J Bunch, Merle B Thomas, Lex B Turner

**Affiliations:** 1 Commonwealth Scientific and Industrial Research Organization, Queensland Bioscience Precinct, 306 Carmody Road, St Lucia, 4067, Queensland, Australia; 2 Queensland Department of Primary Industries and Fisheries, Mutdapilly Research Station, MS 825 Peak Crossing, 4306, Queensland, Australia

## Abstract

**Background:**

The goal of genome wide analyses of polymorphisms is to achieve a better understanding of the link between genotype and phenotype. Part of that goal is to understand the selective forces that have operated on a population.

**Results:**

In this study we compared the signals of selection, identified through population divergence in the Bovine HapMap project, to those found in an independent sample of cattle from Australia. Evidence for population differentiation across the genome, as measured by F_ST_, was highly correlated in the two data sets. Nevertheless, 40% of the variance in F_ST _between the two studies was attributed to the differences in breed composition. Seventy six percent of the variance in F_ST _was attributed to differences in SNP composition and density when the same breeds were compared. The difference between F_ST _of adjacent loci increased rapidly with the increase in distance between SNP, reaching an asymptote after 20 kb. Using 129 SNP that have highly divergent F_ST _values in both data sets, we identified 12 regions that had additive effects on the traits residual feed intake, beef yield or intramuscular fatness measured in the Australian sample. Four of these regions had effects on more than one trait. One of these regions includes the *R3HDM1 *gene, which is under selection in European humans.

**Conclusion:**

Firstly, many different populations will be necessary for a full description of selective signatures across the genome, not just a small set of highly divergent populations. Secondly, it is necessary to use the same SNP when comparing the signatures of selection from one study to another. Thirdly, useful signatures of selection can be obtained where many of the groups have only minor genetic differences and may not be clearly separated in a principal component analysis. Fourthly, combining analyses of genome wide selection signatures and genome wide associations to traits helps to define the trait under selection or the population group in which the QTL is likely to be segregating. Finally, the F_ST _difference between adjacent loci suggests that 150,000 evenly spaced SNP will be required to study selective signatures in all parts of the bovine genome.

## Background

The goal of genome wide analyses of polymorphisms is to achieve a better understanding of the link between genotype and phenotype. The study of a large number of polymorphisms spread across the genome will reveal aspects of the genetic structure of the population, including, in some cases, evidence of adaptive selection across the genome [1,2]. Furthermore, if the individuals in the sample are measured for a range of traits, genome wide association (GWA) studies between the polymorphisms and the trait values can lead to the genetic dissection of traits [3,4]. This applies in particular to complex traits, where genetic and environmental factors combine to produce the phenotype [5-7]. A concordance between SNP showing evidence of genetic selection and association to a trait may help define the phenotype that is under positive selection and may provide some evidence to support the association [8], assuming that samples from populations that segregate the genetic variability in question have been included. There are studies of a few genes that give credibility to the approach [9,10].

Genome wide studies of genetic selection (GWGS) are generally performed separately to GWA despite the potential advantages of combining the information. The reasons for this separation are mainly operational. GWA studies are sampled to study a set of traits, and population stratification is avoided or tightly controlled [11]. Often the studies are restricted to a particular population. Much effort goes into replication of results in an independent sample of the same or similar populations, with a strong effort to perform meta-analyses across data sets. Alternatively, studies of selection are recommended to use the most highly differentiated populations available [12]. These are not necessarily the best populations for gene discovery for any particular trait. One example is the Human HapMap project, which reported the sampling of 3.1 × 10^6 ^single nucleotide polymorphisms (SNP) across the genome [13], studied in three highly divergent population samples. Many genomic regions showed signatures of selection, some identified using more than one method of analysis. However, it is not known what confidence could be placed in these signatures of selection if a different set of divergent populations was used, or if intermediate populations were included.

The Bovine HapMap project was started with the goal of understanding the genetic structure and history of an important livestock species. The expectation was that this study would provide information on the processes of domestication, insights that are not currently available from the study of humans or other animal species. To do this, animals from 19 breeds and two outgroup species were sampled from five continents and all three major branches of cattle [14]. This large number of groups is quite different to the Human HapMap project. The 501 animals were genotyped for a panel of approximately 4 × 10^4 ^SNP and a variety of analyses were performed, including a genome wide assessment of positive selection. Several methods were used to infer evidence of positive selection with many locations identified by more than one method. These methods were 1) the analysis of population divergence using F_ST _[2,15], 2) the analysis of ancestral states in conjunction with extended haplotype homozygosity [16] of derived alleles using the iHS method [17], and 3) the modelling of the distribution of allele frequencies along a chromosome under the expectation of a selective sweep analysed using a composite likelihood ratio (CLR) [18]. However, these methods respond to different signals in the data. The lack of concordance is therefore not evidence of the absence of selection. It is unknown what proportion of these signals would be reproducible in another set of population samples.

This study had two main aims. The first was to compare genome wide signatures of selection found in the Bovine HapMap project with genotypes collected from a similar set of animals for which phenotypes are known [19]. The second was to explore the relationship between signals of selection and associations to traits. The traits were intramuscular fatness, residual food intake and meat yield, which have biological and economic importance. They relate to obvious characteristics of the phenotype and are under artificial selection in cattle. The Australian samples include eight of the breeds used in the Bovine HapMap study. We used F_ST _to study selection partly because 1) it is a robust easily calculated measure with a long history [2,12,15,20,21], 2) the amount of ancestral information on SNP is low in cattle [14], resulting in sparse information for iHS, and 3) the joint minor allele frequency distribution is not known in all breeds making the CLR method complex to implement in cattle.

## Methods

### Samples

Cattle (*Bos taurus *L.) have three major geographic groupings, the European and North Asian taurine, South Asian and East African zebu, and West and South African sanga [22]. Sanga are often referred to as African taurine cattle. Australian samples were collected from 13 breeds of taurine, zebu, zebu-taurine and sanga-taurine animals and known crossbreds (Table 1) after the research plans were submitted for Ethics Approval (ARI 037/2002). Informed consent to use the samples was also obtained from the farmers who owned the cattle. The samples consisted of steers of the taurine British beef breeds of black Angus (ANG), red and white faced Hereford (HFD), the red Shorthorn (SHN), and the white Australian Murray Grey (MGY), a breed heavily influenced by the Angus; the zebu Brahman breed (BRM), which is usually grey but can be red; the composite sanga-taurine Australian Belmont Red (BEL), a composite of the sanga Africander cattle of the San and Khoi of Southern Africa and Hereford and Shorthorn cattle, and the composite zebu-taurine cherry-red American Santa Gertrudis (SGT), a composite of the Brahman and the Shorthorn. The cows of the dairy breeds consisted of the taurine European Brown Swiss (BSW), black and white European/North American Holstein (HOL), the Channel Isle red and white Guernsey (GNS) and tawny Jersey (JER), the red Australian Illawarra Shorthorn (IWS), derived from the Milking Shorthorn, the composite taurine red and white Australian Red Cattle (AUR), consisting of Illawarra Shorthorn, Ayreshire, Scandanavian Red and Red Holstein bloodlines which is being adapted to Australian conditions, and the zebu-taurine composite black and red Australian Friesian Sahiwal (AFS), a milking zebu composite, as well as industry cattle of known crossbred ancestry.

**Table 1:** The number of animals per breed, the breed codes and coding schemes

Type	Breed type	Breed	N	Breed code^1^	data code no^2^	F_ST _group code^3^	Comment
beef	British taurine purebred	Angus	41	ANG	0	0	
beef	British taurine purebred	Hereford	28	HFD	1	1	
beef	British taurine composite	Murray Grey	20	MGY	2	2	
beef	British taurine purebred	Shorthorn	27	SHN	3	3	
beef	sanga-taurine composite	Belmont Red	24	BEL	4	4	
beef	zebu composite	Brahman	21	BRM	5	5	
beef	zebu-taurine composite	Santa Gertrudis	28	SGT	6	6	
dairy	European taurine purebred	Brown Swiss	4	BSW	7	7	
dairy	European taurine purebred	Holstein	52	HOL	8	8	
dairy	European taurine crossbred	Brown Swiss Holstein	26	BHX	9	7	
dairy	Channel Isle purebred	Guernsey	4	GNS	10	9	
dairy	Channel Isle purebred	Jersey	10	JER	11	9	
dairy	Channel Isle European crossbred	Guernsey Holstein	2	GHX	12	9	
dairy	Channel Isle European crossbred	Holstein Jersey	9	HJX	13	9	
dairy	British taurine purebred	Illawarra Shorthorn	8	IWS	14	10	
dairy	British European Scandinavian crossbred	Australian Red	54	AUR	15	10	3breed
dairy	European crossbred	Brown Swiss cross	5	BSWX	16	7	3breed
dairy	European crossbred	Holstein cross	3	HOLX	17	8	3breed
dairy	Channel Isle crossbred	Jersey cross	4	JERX	18	9	3breed
dairy	zebu-taurine composite	Australian Friesian Sahiwal	5	AFS	19	999	excluded from F_ST_
extra	many breeds	repeats and unknown crossbred	10	NA	999	999	

^1^Breed Code is the 3-letter code for each breed, ^2^data code no is the numerical code for each breed in the analysis, and ^3^F_ST _group code is the grouping used for the analysis of positive selection. Code values of 999 mean coded as missing data.

The beef cattle samples and their trait measurements have been described previously [19,23,24]. In short, the beef sample consisted of 189 individuals from 142 sires chosen to avoid close relatives. There were one to four offspring per sire and a median of one offspring per sire. This sub-sample was chosen from a larger group of 1472 animals in half-sib pedigrees representing 308 sires. The detailed description of the method of choosing the sub-sample has been published [19]. The dairy samples were collected as part of an industry survey of cattle tick burden of 2494 cattle in North Eastern Australia. The sub-sample of 189 animals consisted of offspring of 138 sires and 174 dams, with a median of one offspring per sire and a range of 1–4 offspring per sire. They were chosen for genotyping from 5 properties where tick counts had been obtained over several seasons. The animals were chosen to be from as many breeds and sire lines as possible, irrespective of their tick burdens. They were also chosen because they had full pedigree records in the Australian Dairy Herd Information Service (ADHIS) database with full milk yield performance. Because of this, obtaining equal numbers for each breed with such a specification was not possible, because the breeds do not have equal representation in Australia. Aliquots of 200 μL of blood were processed to DNA using the QIAamp DNA mini kit using the manufacturer's instructions (QIAGEN GmbH, D-40724, Hilden, Germany).

The Bovine HapMap sample, genotypes and quality control have been described previously [14]. In brief, samples were obtained for the taurine beef breeds Angus, Charolais, Hereford, Limousin, Piedmontese, Red Angus, and Romagnola; the taurine dairy breeds Brown Swiss, Guernsey, Holstein, Jersey, and Norwegian Red; the zebu Brahman, Gir, and Nelore; the sanga N'Dama breed; the zebu-taurine composite breeds of Beefmaster and Santa Gertrudis; and the sanga-zebu composite Sheko breed. These samples were genotyped for approximately 4 × 10^4 ^SNP derived from the Bovine Genome Sequencing Project. Each breed sample consisted of 24 animals including two trios of sire, dam and offspring. The rest were animals as unrelated as possible using a pedigree analysis. The exceptions were the Holstein, the Limousin and the Red Angus, which had samples of 53, 42 and 12 respectively. For F_ST _calculation the offspring were excluded. The correlation of F_ST _with and without these offspring across all loci was r = 0.997. A link to the SNP can be found at  associated with the Baylor College of Medicine and the description of the SNP is subject to the Fort Lauderdale agreement [25]. The order and distance of SNP along the bovine genome was taken from the current assembly in Genbank (Btau4.0).

### Genotyping

The genotyping of the Australian sample was performed using the MegAllele™ Genotyping Bovine 10 K SNP Panel [26], a fully described set of SNP, by ParAllele Inc. on an Affymetrix GeneChip Scanner 3000, yielding an average spacing of 325 kb between SNP. Further details of the SNP can be found at the link . The bulk of the SNP on the SNP array were obtained by comparing the genome sequence of the Hereford animal to the partial sequence of the Holstein (72.4%) and the Angus (15%) animal, with 7.5% cSNP (coding SNP) obtained from the Interactive Bovine in silico SNP database [27], and the rest from the partial sequence of the Limousin (3.1%) and the Brahman (2%) animal. The major difference in breed origin of SNP in the two SNP sets is an increased proportion of SNP obtained by comparing the genome sequence of the Hereford animal to the partial genome sequence of the Norwegian Red (33%), the Brahman (13%) and the Jersey (2%) animal, with a consequent decrease in the percentages of SNP obtained from an animal of another breed [14]. In summary, the SNP in both SNP sets are mainly the pairwise differences between a taurine beef and dairy animal, with either a small or a moderate percentage of SNP that are differences between a taurine and a zebu beef animal. The beef and dairy samples had been genotyped separately so the combined data set was rescored by ParAllele to ensure that genotypes were consistent. Data quality assurance was investigated using duplicate samples (unknown to the genotyper) and the genotyping of a selection of loci using two alternative technologies. Repeat genotyping of the same individual showed a 99.72% concordance rate. All samples with more than 10% missing data were excluded and then all loci with more than 10% of missing data were excluded. The rationale for this is that DNA that is not of high quality will be more likely to have incorrect genotype calls and have more missing data. The same will apply to SNP with poor assays, which are more likely to have false or indeterminate calls, and so are also more likely to have missing data. The rejection of 10% was not arbitrary; the number of loci excluded was plotted against percent missing data and the cut off point was determined as the point in round numbers along the curve where the rate of change in the number of excluded loci became linear with every increased percent of missing data. This left 8859 SNP out of the panel of 9276 scored SNP. These SNP were tested for departures from Hardy-Weinberg equilibrium within breed, and 4.99% of comparisons were significant at the 5% level.

### Analysis

To determine the clustering of individuals in this study, a principal component analysis (PCA) was performed on the individual genotypes of the 378 animals using the 1604 SNP that had no missing data. The data were analysed using a covariance matrix in SPLUS [28-31]. Full records, without missing data, were used because individuals with missing data may appear as outliers [30]. The reduction in the number of loci has the benefit of reducing the correlation between genotypes due to linkage disequilibrium (LD); the average gap between adjacent loci increased from 357 kb to 1.74 Mb and the number of SNP separated by less than 10 kb dropped from 2462 to 187. The first principal components (PC) were highly significant and these were plotted against each other, encoding each coordinate with the breed or crossbred identity of the animal as contained in the database. During the analysis we found an Angus and a Murray Grey individual clustered with the zebu-taurine composite animals. Those animals were found to have a mismatch between the DNA sample and breed designations in the Beef CRC database. They were excluded from the F_ST _analysis although we do not expect that the exclusion would make much difference to the analysis because of the small numbers involved and the amount of variation explained by each principal component. The clustering was performed for two reasons. Firstly, lumping of breeds was necessary in the dairy animals because some of the sample sizes were small (*N *< 10). It has been shown that it is best to avoid small samples and to use samples of equal size when comparing estimates of F_ST _[12]. Having similar groups will generate less variable estimates of F_ST_, while lumping groups that have divergent genotypic frequencies should act to reduce F_ST_. Nevertheless, it is the distribution of F_ST _across the genome not the absolute value that is important in identifying evidence of positive selection [2]. Secondly, the clustering identifies the most divergent breed samples in a convenient graphical output. Comparison of the most divergent two to four groups will encompass most of the variability captured by F_ST _[12]. The analysis of a common set of divergent breeds will allow a baseline comparison between the Bovine HapMap and this study.

To determine a genome wide pattern of positive selection, and to compare this to the Bovine HapMap sample, the F_ST _at each locus was calculated [32] in the same way as in the Bovine HapMap sample. All F_ST _values in this study are per locus values. Means and standard deviations were calculated not to find an unbiased average across all the data but to measure the dispersion of per locus F_ST _in the sample. SNPs that were genotyped in all breeds were used. F_ST_, the average heterozygosity and average allele frequency for each SNP were plotted (Additional File 1). Examination of this figure allows one to see whether the F_ST _values were likely to be affected by significant patterns in genotypic frequencies. The F_ST _values were then plotted against genome location and values were averaged over 8 SNP in a sliding window, as in the Bovine HapMap project. Signatures of selection can be recognized when adjacent SNPs all show high F_ST _[2], due to the hitch-hiking effect [33], implying divergent selection between breeds, or where adjacent SNPs all show low F_ST_, implying balancing selection between breeds [15,21]. Several comparisons were made, varying the breeds and the SNP that were included. The baseline comparison was for the three breeds Angus, Brahman and Holstein, which are the most divergent samples in the PCA in this study, and which were sampled in the Bovine HapMap study. The Pearson correlation coefficient was calculated between the F_ST _values from this study and those from the Bovine HapMap study. The F_ST _values in the top and bottom 2.5% of the distribution in both data sets were compared. To determine whether these loci were significant, the confidence limits for F_ST _were calculated for both data sets. To obtain confidence limits for the per locus value of F_ST_, 1,000 bootstrap samples were taken each consisting of 1,000 F_ST _values sampled at random from the loci within each data set. The average F_ST _was calculated for each bootstrap sample and the standard deviation of the resulting average F_ST _values was used to set confidence limits [34,35]. To describe the relationship between the location and diversity along a chromosome, the difference between the F_ST _(δF_ST_) of pairs of immediately adjacent SNP (SNP pairs) was compared to the distance in base pairs between them. Each interval between SNP pairs was used once. SNP pairs were binned into those below 1 kb, then into 10 kb bins up to 100 kb, and then into 100 kb bins up to 1.2 Mb. The mean δF_ST _and standard error were plotted against mean distance between the SNP pairs of each bin.

The additive and allele substitution effects for the traits residual feed intake (RFI), percent meat yield (yield), and percent intramuscular fat (IMF) in beef cattle were calculated as previously described and results published elsewhere [19,36,37]. In brief, residual trait values were obtained from a mixed model that incorporated fixed environmental effects as well as the random effect of breed (herd of origin) and sire, thereby accounting for possible stratification in the data, using ASReml [38]. The additive effect (*a*) is half the distance between the two homozygotes, the dominance effect (*d*) is the difference between the heterozygotes and the average of the two homozygotes, and the allele substitution effect (α) = *a *+ *d*(q-p) where p+q = 1 and p, q are the allele frequencies [39]. Standard errors were estimated using 1000 bootstrap samples [40]. In this study the estimates for beef traits were reported within taurine breeds (TEM temperate ANG, HFD, MGY, SHN) and within zebu plus tropical composites (TRO tropical BEL, BRM, SGT). Breed type was used because the sample sizes within each breed would be too small for meaningful analysis. As the trait values were adjusted for the effects of breed and contemporary group, a combined analysis should not produce spurious results. These results were searched using the list of SNP that had extreme F_ST _values.

The number of association tests with high extreme F_ST _versus low extreme F_ST _was compared to the goodness of fit expectations for the same sample size using a chi-square test. The expected frequency was taken as the ratio of the observed number of SNP in the low versus the high extreme.

## Results

**Figure 1:**
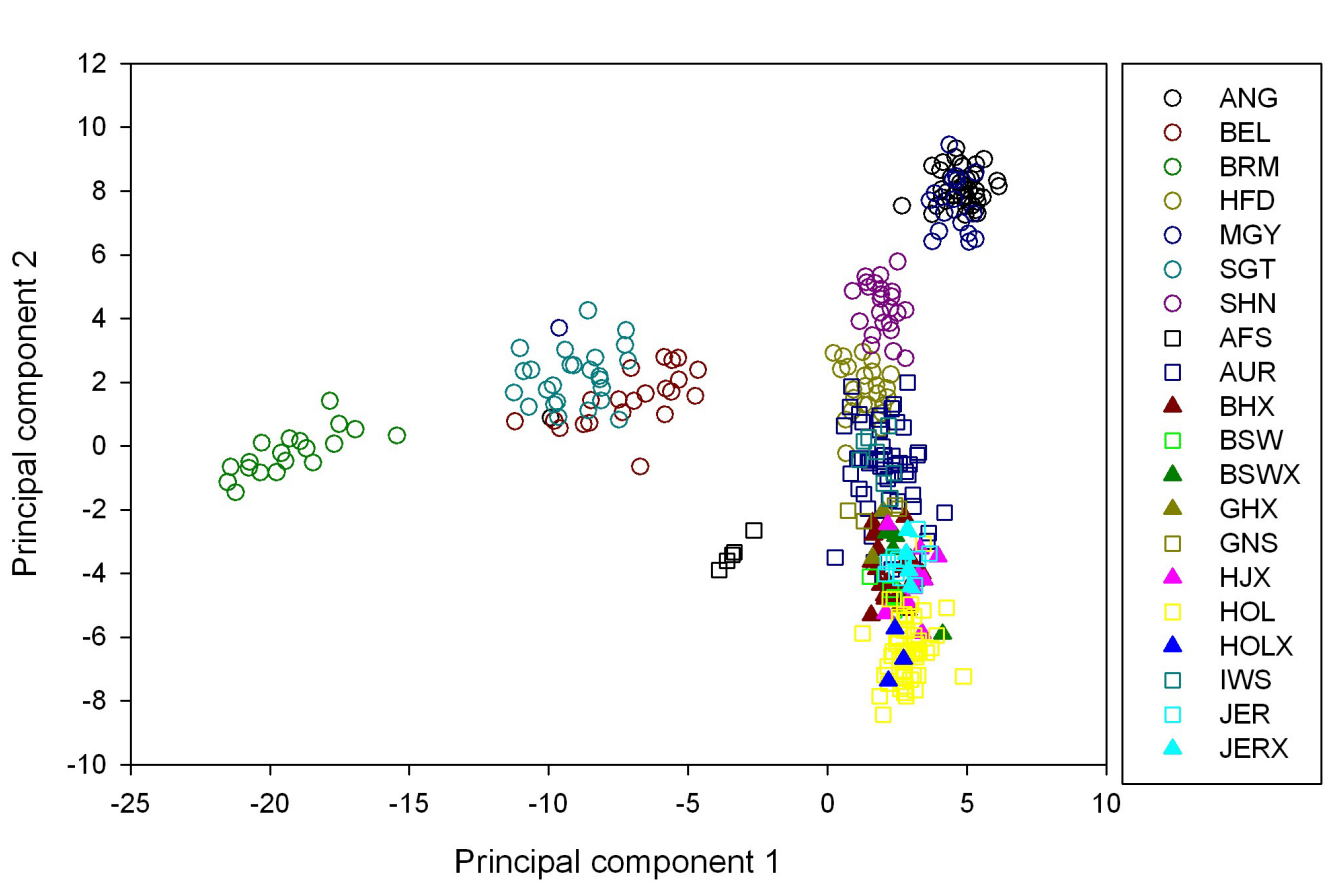
**Animals clustered on the basis of principal components of genotypic variation**. The crossbred dairy samples cluster mainly with the Brown Swiss and Channel Island breeds.

Before calculating F_ST_, we used a PCA to determine how the animals should be allocated to groups and the degree of divergence between samples. Using the individual genotypes as the data in the PCA (Figure 1), PC1 explained 6.1% and PC2 explained 3.7% of the variance. We found that PC1 separated out the zebu Brahman breed from the taurine breeds. Animals with known recent part zebu ancestry, such as the Santa Gertrudis and the Australian Friesian Sahiwal, were also separated from the taurine breeds along the PC1 axis, although not to the same extent as the Brahman. The Belmont Red, which in principle does not have recent zebu ancestry but in practise may have a small percentage of Brahman ancestry, was also separated along PC1 to the same extent as the Santa Gertrudis. The taurine breeds were partly separated along PC2. In general the animals of one taurine breed clustered together, but the clusters partly overlapped those of other breeds located near by. Animals of the Holstein dairy breed were located at one end and animals of the Angus beef breed were located at the other end of the spread of breeds along PC2. The Hereford breed occurred at the intersection of the axes of PC1 and PC2. The locations of the Angus, Hereford and Holstein are consistent with the process of SNP discovery, where most SNP in the 10 K SNP set were obtained by comparing the Holstein and Angus to the Hereford. The complete overlapping of the Angus and Murray Grey was expected given the role of the Angus in the development of the Murray Grey. The slight separation between the Angus and Murray Grey breeds and the other taurine breeds may be due to the absence of a full range of breeds rather than any particular distinctness of the Angus and Murray Grey from the others. The breeds that were furthest apart on these two axes in this study are the Angus, Brahman and Holstein. Based on the PCA, the dairy animals were lumped into four groups of reasonable size for the F_ST _analysis, as indicated in Table 1. The Australian Friesian Sahiwal were excluded from the F_ST _calculations because of their small sample size and lack of affinity to other breeds.

Comparing this plot of PC1 and PC2 to the plot of the first two components in the Bovine HapMap study, PC1 also separated the zebu from the taurine breeds in that study while PC2 partly separated the taurine breeds as a series of partly overlapping clusters. PC1 clearly separated the one sanga breed, the N'Dama, from the zebu breeds, to the same distance as that between the zebu and taurine breeds. PC2 clearly separated the taurine from sanga breeds. There were no purebred sanga animals among the Australian samples. The one major difference was the Hereford breed in the Bovine HapMap study, which was located well away from the other breeds as a loose, flat cluster. Of the other taurine breeds in the Bovine HapMap study, the Angus and Holstein were the furthest apart in the plot of PC1 and PC2.

To characterise the differences in per locus F_ST _between this and the Bovine HapMap study, we compared several groupings of breeds and loci (Table 2). There were minimums of 32224 SNP in the Bovine HapMap data and 8644 SNP for the Australian data with a per locus F_ST _value when all populations were used. The Australian data showed lower per locus F_ST _values than the Bovine HapMap data with a lower amount of dispersion around this mean value. In both studies, the mean per locus F_ST _was larger when only the three divergent breeds Angus, Brahman and Holstein were used. In the Bovine HapMap data this was so whether all loci or only the loci in common to both studies (N = 7298) were used. The difference in mean per locus F_ST _value between the Australian sample and the Bovine HapMap sample was less when only the three divergent breeds were compared. Although these mean differences are small, they are all significant because of the large samples of loci used. The dispersion of F_ST _values was also greater in the three breed comparisons than when all breeds where used. To determine if removing some breeds would make a difference to the estimates, we removed the two African breeds, N'Dama and Sheko, and the two zebu breeds, Gir and Nelore, from the Bovine HapMap data. Removing the N'Dama and Sheko removed animals that were highly divergent on PC2 of the Bovine HapMap data, leaving only the partly overlapping European breeds. Removing the Gir and Nelore reduced the number of zebu breeds to one. This made the breed composition more similar in the two studies. It also still included the Angus, Brahman and Holstein. For all loci in the Bovine HapMap data the average F_ST _= 0.126, S.D. = 0.0722 (N = 32470). This showed a reduction in the per locus F_ST_, of nearly half the difference between the mean per-locus F_ST _value of the full Bovine HapMap and Australian samples. This reduced set of breeds still showed the lower dispersion in per locus F_ST _values found in the full sample of breeds compared to the three breed estimates.

**Table 2:** The per locus F_ST _values in different data sets

Comparison		Australian Samples	Bovine HapMap common loci	Bovine HapMap all loci
All breeds	mean	0.094	0.141	0.151
	S.D.	0.0540	0.0633	0.0795
Three breeds	mean	0.126	0.154	0.172
	S.D.	0.1095	0.1255	0.1447

**Figure 2:**
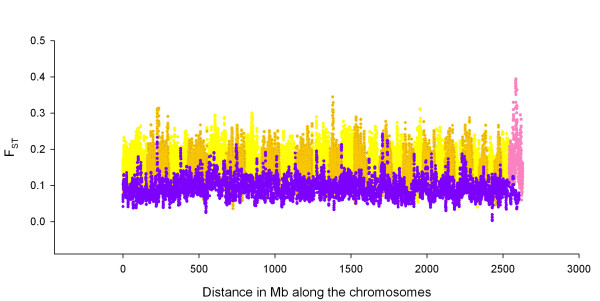
**Genome wide picture of positive selection**. The distribution of F_ST _for all breeds calculated in a sliding 8 SNP window along the chromosomes, with the Bovine HapMap values plotted on the same axes as the values calculated in the Australian cattle sample, for all loci in each study. The F_ST _values of the Bovine HapMap sample are noted in yellow and ochre for odd and even autosomes respectively with the X chromosome in magenta. The F_ST _values of the Australian cattle sample are noted in purple. Extremely high values represent likely instances of divergent selection and extremely low values represent likely instances of balancing selection.

To determine whether the global pattern of F_ST _across the genome was the same in both this and the Bovine HapMap study, the windowed F_ST _was plotted against location in the genome (Figure 2). There were obvious differences in the locations of the major peaks. The difference in height along the ordinate was consistent with the average difference found for F_ST _(cf. above). In particular, locations on chromosomes 7, 10, 12, 14 and the X were obviously different. The windowed F_ST _values between the two data sets at each locus were correlated with r = 0.346, N = 7298, P = 0. This indicated that genome location explained 12.0% of the variance in that comparison.

**Figure 3:**
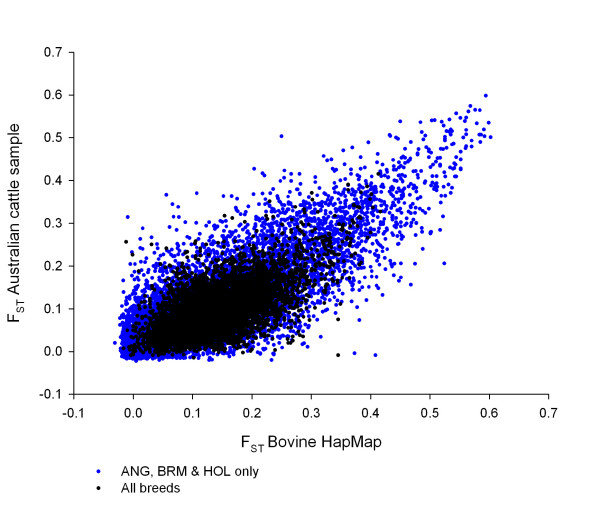
**The F_ST _values in the sample of Australian cattle plotted against those from the Bovine HapMap study**. The values for all breeds are in black and the values for the Angus, Brahman and Holstein are in blue.

The relationship between genome location and F_ST _was explored in more detail by using subsets of breeds and subsets of loci to determine which had the more important influence on whether a signal appeared in a particular location. Using only the loci in common with all breeds in both data sets, the un-windowed F_ST _values showed a correlation of r = 0.615 (Figure 3). Due to the number of common loci or common windowed points in common, N = 7298, all of the correlations reported below are highly significant, with P << 0.0001. For the subset of three divergent breeds the un-windowed F_ST _values showed a correlation of r = 0.787, or 63.5% of the variance in F_ST _(Figure 3). The three breed comparison had a broader range of F_ST _values. Comparison of the three breed to the all breed correlation for un-windowed F_ST _values showed that changing the breed composition in this experiment resulted in a 40.4% decrease in the amount of the variance in F_ST _explained across the genome.

To determine the effect of the specific loci on the distribution of F_ST_, we compared the windowed F_ST _values in this study to the windowed F_ST _values in the Bovine HapMap study. The windows are for 8 adjacent loci so the composition and density of loci contributing to each reference point along the genome differed in the two data sets. For all loci the correlation was r = 0.346 for the all breed F_ST _values and r = 0.391 for the three breed F_ST _values. Comparing the correlation of three breed windowed F_ST _values of the Bovine HapMap data and the Australian data to the correlation of the un-windowed values for those data shows a reduction in the variance explained of 76% (r = 0.391 vs r = 0.787). Comparing the all breed windowed to un-windowed F_ST _values in the same way shows a reduction in the variance explained of 68% (r = 0.346 vs r = 0.615). This reduction was smaller than the three breed comparison but the full breed comparison includes not only differences in SNP between studies but also differences in the number and composition of breeds.

To determine the importance of differences in SNP density between the two studies, windowed F_ST _values for BTA 6, 14 and 25 were compared to the other chromosomes. These three chromosomes have 2–3 times higher density than the other chromosomes in the Bovine HapMap data, which in their turn have a 2–3 times higher density in the Bovine HapMap data than in the Australian data. For all breeds, comparing the Australian to the Bovine HapMap data, the correlations were r = 0.382 for comparisons of BTA6, 14 and 25 combined and r = 0.344 for the other chromosomes combined. If the loci that are windowed are only the common ones between the two studies, that is, no difference in density, then the correlation between windowed F_ST _values is r = 0.640, essentially the same as the un-windowed values (r = 0.615).

We calculated the confidence interval for the per locus F_ST _to be considered significant using bootstrap sampling. The 99.9% confidence interval for the per locus F_ST _in our data was 0.094 ± 0.062 while the confidence interval in the Bovine HapMap data for the same loci was 0.141 ± 0.121. In the Australian sample, the top 2.5% corresponded to a threshold F_ST _= 0.224 and the bottom 2.5% corresponded to a threshold F_ST _= 0.015, both outside the confidence interval for that dataset. In the Bovine HapMap data, the top 2.5% corresponded to a threshold F_ST _= 0.284, which is outside the 99.9% confidence interval and the bottom 2.5% corresponded to a threshold F_ST _= 0.039, which is outside a 99% confidence interval for that dataset. There were 94 loci that had an F_ST _above the upper thresholds in both data sets, or 1.28% of 7298 SNP. There were 35 loci that had an F_ST _below the lower thresholds in both data sets. For the SNP above the threshold in both data sets, the 94 SNP were located in 71 genomic regions of 1 Mb containing one or more SNP with high F_ST _(Additional File 2). For the SNP below the threshold in both data sets, almost none of those SNP were close to another SNP with low F_ST_. Some of the low F_ST _values are negative, which may occur when estimating an F_ST _value near zero, but which may also occur in some cases where the expected variance calculated from the average allele frequency for the entire sample is sufficiently less than the sample variance in allele frequencies across populations, or which may occur in some cases where the heterozygote frequencies within populations are higher than expected given the allele frequencies.

**Figure 4:**
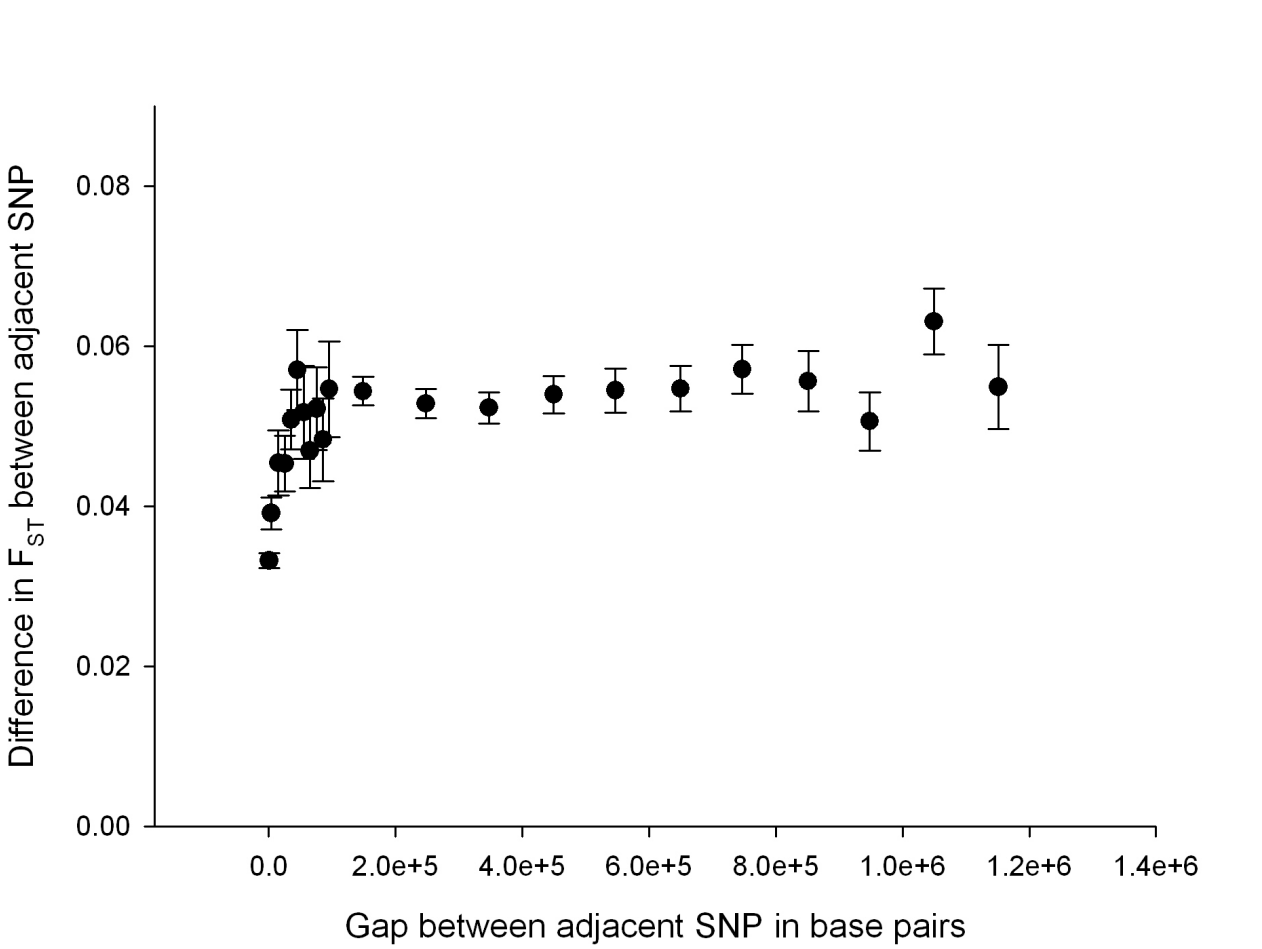
**The increase in difference in F_ST _between adjacent SNP as the distance between adjacent SNP increases in the Australian cattle sample**. The mean value of each bin is plotted with its standard error.

The average δF_ST _between the SNP pairs was strongly influenced by the distance between them. The δF_ST _increased quickly with distance and reached a plateau after approximately 20 kb (Figure 4). The average δF_ST _associated with the bin sizes of <1 kb, 1–10 kb and 10–20 kb were all significantly different from each other, and each increase in average distance was accompanied by an increase in average δF_ST _until the plateau was reached. The plot showed broader standard errors for comparisons in the 20–100 kb region, but this was due to smaller numbers of SNP separated by those gaps. For example, there were 2061 snp pairs separated by < 1 kb in that plot, 402 pairs separated by 10–20 kb, and between 73 and 129 pairs in each of the 10 kb bins from 20–100 kb. The plot shows that the bins between 20–100 kb had similar means. For the combined bin 20–100 kb, corresponding to a mean separation of 56.7 kb between SNP pairs, the mean δF_ST _= 0.051, SE = 0.0017, *N *= 734.

Fourteen of the 129 SNP with extreme F_ST _in both data sets had an effect on one of the three traits (RFI, yield and IMF), six of which had an effect on more than one trait (Table 3). The same homozygote in one of the six SNP increased both yield and IMF, and the same homozygotes in the remaining five SNP increased both IMF and RFI. The effects that were significant for more than one trait were all in the same breed type. Three of the six SNP were located to one small region of bovine chromosome 2. Counting these three as one independent locus, four (ie 6-2) of the 12 (ie 14-2) independent SNP had effects on more than one trait, or 33%. Only 13.9% of the SNP in the entire experiment that had an effect on RFI also had an effect on IMF and only 7.0% that had on effect on yield also had an effect on IMF. This difference in frequency, while large, was not significant (χ^2^_1 _= 3.78 n.s.). All of these SNP had F_ST _values greater than the upper threshold, representing divergence between the breeds. The observed frequency of 0 low F_ST _versus 14 high F_ST_, compared to the expected frequency of 35:94, was significant (goodness of fit χ^2^_1 _= 5.21, *P *< 0.05). These loci all had much larger F_ST _values in the three breed sub-sample than in the full breed sample.

**Table 3:** Trait associations and high F_ST _values

Locus	Chr	Position	Group	N	p_0_	V_r_	a	SE_a_	α	SE_α_
Multiple traits										
RFI										
rs29019351	2	64740286	TEM	104	0.91	0.0370	0.654	0.214	-0.169	0.219
rs29019352	2	64740428	TEM	104	0.09	0.0370	-0.654	0.209	0.169	0.223
rs29021800	2	64792978	TEM	102	0.08	0.0412	-0.653	0.209	0.259	0.223
rs29025811	5	51065596	TRO	65	0.76	0.0405	-0.401	0.191	-0.374	0.206
rs29010304	28	24504312	TRO	65	0.28	0.0659	0.332	0.218	0.487	0.239
Yield										
rs29015041	6	88160023	TEM	58	0.16	0.0517	-0.900	0.320	-0.555	0.333
IMF										
rs29019351	2	64740286	TEM	99	0.91	0.0212	0.554	0.223	0.354	0.243
rs29019352	2	64740428	TEM	99	0.09	0.0212	-0.554	0.222	-0.354	0.250
rs29021800	2	64792978	TEM	97	0.09	0.0190	-0.550	0.229	-0.282	0.245
rs29025811	5	51065596	TRO	62	0.75	0.0808	-0.512	0.159	-0.344	0.162
rs29015041	6	88160023	TEM	99	0.22	0.0380	-0.354	0.182	-0.439	0.193
rs29010304	28	24504312	TRO	62	0.29	0.0972	0.228	0.162	0.440	0.188
Single traits										
RFI										
rs29021601	11	39253966	TEM	104	0.84	0.0403	-0.363	0.201	-0.483	0.209
rs29019566	15	18646517	TEM	102	0.93	0.0253	0.505	0.210	-0.115	0.214
rs29026034	16	23320981	TEM	103	0.83	0.0399	-0.680	0.188	-0.286	0.179
Yield										
rs29011076	4	11913823	TEM	58	0.69	0.1102	0.527	0.374	0.873	0.341
rs29013771	4	54568493	TEM	53	0.13	0.0454	0.650	0.331	-0.317	0.378
rs29026560	9	47205531	TEM	58	0.28	0.0505	1.038	0.327	0.733	0.324
IMF										
rs29012117	13	28925713	TRO	62	0.57	0.0814	-0.435	0.177	-0.410	0.177
rs29021963	16	31068223	TRO	57	0.62	0.1100	-0.469	0.177	-0.392	0.242

Locus dbSNP identifier, Chr chromosome Position in bp, Group breed type, temperate or tropical, N sample size, p_0 _is the allele higher up the alphabet, V_r _is the residual variance explained by the genotypes, *a *is the additive effect, SE_a _is the standard error of a, α is the allele substitution effect, SE_α_is the standard error of α.

RFI residual food intake in kg per day, Yield is the meat yield of the animal as a percentage, and IMF is the intramuscular fat percentage

The region on BTA2:64.7 Mb that showed effects on two traits was examined in greater detail. There are eight SNP in this region separated by a total of 83.3 kb set in a region containing 31 SNP separated by a total of 9.86 Mb. Thirteen of the loci (Table 4) showed a significant additive or allele substitution effect on RFI or IMF in either temperate, tropical or the combined sample. Six of the 13 SNP showed an association but the F_ST _value was within the confidence interval for the data set. Nine of the 13 SNP showed an association and had average allele frequencies of between 0.1 and 0.9 across all breeds. Only the three SNP with the highest F_ST _values had effects on both traits.

**Table 4:** Significant associations to traits across 10 Mb of BTA2

Locus	BTA2 position	P_ave_	F_ST_	RFI	Type_RFI_	IMF	Type_IMF_
rs29021692	60071311	0.625	0.098			2.782	TRO, TTL
rs29020674	60485596	0.097	0.253			2.513	TRO^α^
rs29017559	62324132	0.155	0.068			1.985	TTL
rs29019351	64740286	0.866	0.313	3.057	TEM	2.483	TEM
rs29019352	64740428	0.115	0.314	3.130	TEM	2.490	TEM
rs29020718	64778749	0.096	0.204	2.203	TEM		
rs29020715	64778938	0.904	0.204	2.111	TEM		
rs29021800	64792978	0.134	0.312	3.122	TEM	2.403	TEM
rs29021802	64797701	0.043	0.179	1.986	TTL		
rs29015936	65535887	0.853	0.034			2.229	TTL
rs29015937	65535915	0.464	0.100	1.986	TRO		
rs29024553	68241372	0.278	0.070			2.034	TTL
rs29010833	69818303	0.680	0.041			2.267	TTL

P_ave _is the average allele frequency of p_0 _across the breeds, RFI is the ratio between the additive effect and its standard error, Type_RFI _is the type of cattle in which the effect was estimated, IMF is the ratio between the additive effect and its standard error, Type_IMF _is the type of cattle in which the effect was estimated. TEM temperate, TRO tropical, TTL total. TRO^α ^means the effect is the allele substitution effect.

## Discussion

In this study we report a high level of concordance between the F_ST _values calculated from this study and the Bovine HapMap data for the same SNP and breeds. When the same divergent breeds, namely, the Angus, Brahman and Holstein, are compared, the correlation in per locus F_ST _values between the Australian data and the Bovine HapMap data was high. In both data sets we found that calculating F_ST _using a large number of breeds resulted in per-locus F_ST _values that were not as broadly dispersed around a mean value as those F_ST _values calculated using only the three divergent breeds. This suggests that using many breeds or population groups acts to reduce the variability in estimated per-locus F_ST _values.

However, there are major differences between the two studies in which regions of the genome show the most divergence when all breeds and all loci are used. Many of the strongest signals of selection in the Bovine HapMap data were not found in our data, and the pattern of the signal across chromosomes was visibly different. The similarity between the F_ST _values declined significantly when all breeds were used, amounting to 40% of the variance compared to when only the three divergent breeds were used; there are only eight breeds in common to both studies. The lower correlation with different breeds may suggest that each study had identified signals of divergence particular to the genetic history of those breeds, some of which may be due to selection. The relationship between F_ST _values also decreases substantially when the composition of SNP differed, amounting to 76% of the variance in these data sets when the three divergent breeds are compared.

As there were only 7298 loci in common to both studies, a large proportion of the difference in F_ST _values between two studies will be due to different SNP compositions in the data. For the common loci, the identity and density are the same, and, because the common loci form most of the loci in the Australian set, density and identity are confounded when there is no difference in density between the two studies. Nevertheless, above that baseline, the three higher density chromosomes of the Bovine HapMap data (BTA6, 14 and 25), showed essentially the same relationship to the Australian data as the rest of the data set. This suggests that SNP identity was more important than density in explaining the differences between the two studies. The similarity between the F_ST _values between the two datasets declined significantly when all breeds and SNP were used, amounting to 68% of the variance in that comparison. This is less than the 76% when only the Angus, Holstein and Brahman breeds of the two data sets are used. This difference suggests an interaction between breeds and SNP. The composition of SNP in the two panels is neither of uniform ascertainment nor of exactly the same ascertainment as each other. It is not uniform because SNP were primarily generated by comparing a small number of animals, each of a different breed, to the Hereford sequence, and it was different, because there were slightly more breeds used to define SNP in the Bovine HapMap data and a much larger percentage of zebu SNP [14].

The first two axes of the PCA separated breeds in a similar way in the Australian and the Bovine HapMap data with one major exception. The similarities are that the first axis clearly separated zebu from taurine and the second axis partially separated meat and dairy within European breeds in both data sets. The first axis also clearly separated zebu from purebred sanga in the Bovine HapMap data, which were at the same co-ordinates on PC1 as the European taurine group. The second axis in the Bovine HapMap study clearly separated the sanga (African taurine) from the European taurine. The differences between European cattle were minor compared to the differences between European and African cattle. Based on the Bovine HapMap data, for diversity studies, a minimum three breed panel would probably have to consist of one taurine, one sanga and one zebu breed. Sanga breeds are not common in the developed world. For breeds that are commonly available one could identify the Angus, Brahman and Holstein as a minimum group for studies of breed or locus divergence based on the similarity between the two studies. However, the greater difference between Angus and Holstein compared to other European breeds may be a consequence of the SNP ascertainment, and further study might be needed before a minimum panel of common breeds could be specified. The major difference between the two studies was that we found that the reference breed for SNP discovery, the Hereford, was located exactly where it was expected, at the intersection of PC1 and PC2, because all breeds would in effect be compared to it (cf. above). This location of the Hereford cluster differs substantially from the analysis reported in the Bovine HapMap study. There the Hereford formed a flattened outlier group well separated from the other breeds. In that report, when only the loci originating from the Jersey breed were analysed in the PCA, the Jersey animals were clustered outside the main group of taurine animals. This may indicate that the shifting clusters could be a generic factor associated with the analysis. While we have not explicitly modelled causes for the difference by performing a PCA on the Bovine HapMap data ourselves, if that explanation is correct then it may suggest that the use of a very large number of loci may lead to the unusual clustering of animals that acted as the source of the polymorphisms.

Higher densities of SNP will be required to study genome wide signatures of selection in cattle. LD causes correlation between genotypes [41], so F_ST _values that are more similar at a close distance are likely to be similar due to LD. Our results show that SNP separated by more than 20 kb have δF_ST _the same as those separated by more than 1 Mb. This is consistent with the decay in LD as measured by *r*^2 ^for these [19] as well as the Bovine HapMap data [14], where *r*^2 ^reaches a lower asymptote at around 20–30 kb. These results suggest that for cattle, F_ST _values that are consistently high or low over distances much greater than 20 kb are likely to be due to factors other than baseline LD, and presumably some of these will be due to positive selection [16]. The density of markers required to demonstrate similarity beyond 20 kb implies SNP spaced at less than 20 kb, so that the relationship of δF_ST _and distance between SNP can be quantified. It will require at least 150,000 evenly spaced SNP to quantify genome wide signals in cattle. As SNP are not evenly spaced in current SNP chips, it would require several fold more SNP than that to explore all regions at that minimum density.

The list of the SNP with the most divergent F_ST _values is a resource for the further investigation of the effects of positive selection on the bovine genome. Comparing ends of the distribution in two studies does not necessarily mean that those values are more divergent than expected [34]. Due to the correlation of genotypes in two samples from the same population, very similar F_ST _values can be expected unless there are differences in the populations or errors in the way the genotypes are collected. Instead, the loci in this list have F_ST _outside the bootstrap confidence limits [34] for both data sets. This indicates that they are unlikely to be merely the result of stochastic events during the evolution and domestication of cattle. One would predict that the process of domestication and breed formation would result in more divergent selection than balancing selection. This is because breeds would be formed and then selected for particular characteristics. Breeds are selected to be different to each other. We found that there were more than twice as many divergent than convergent loci when the Australian and Bovine HapMap data were compared to each other, which is consistent with that prediction. These SNP represent genomic regions that should be of general interest, because they represent the overlap of two studies that do not have the same breed composition. Many of these loci are located within 1 Mb of each other. One question that greater SNP density will be able to answer is the detail of differences in F_ST _around these SNP. That should help define groups of divergent or convergent F_ST_, which in turn would help to define the size and shape of these prospective signatures of positive selection.

There appears to be significantly more loci with very high F_ST _that are associated with RFI, yield and IMF than those with very low F_ST_. IMF is strongly correlated to overall fatness of the animal and is negatively correlated with yield, larger RFI values have a positive relationship to IMF and a negative relationship to measures of muscle area in cattle, while the mean values for these traits show large differences between breeds [42-44]. Yield, fatness and efficiency have been valued for hundreds of years in some breeds [45]. Much of this divergence is expected to be the result of artificial selection, because major differences in size and shape are known to have occurred during the domestication of cattle when compared to the extinct wild aurochs [46]. These breeds each have a specification of what the animals should look like and how they should perform that includes yield and fatness. One would therefore expect that some of the loci associated with these traits should show strong differences in genotype frequency between breeds, which would show up in a calculation of F_ST_. It is interesting that of the SNP with divergent F_ST _five out of six of those with effects on more than one of these traits have effects that are consistent with the known correlations between RFI, yield and IMF.

Examination of a region with enough density of SNP shows that association to the trait is not just found for markers with high F_ST_. Associations are found for SNP that do not have highly skewed allele frequencies, and associations are not just limited to a particular subsection of the sample. This suggests that these results are not merely a spurious intersection of high F_ST _and trait association. Functional evaluation of these and other SNP will be needed to show where the causative mutations lie in this region. Full genetic evaluation of these regions will require more SNP, larger samples and more breeds. Nevertheless, our data show that signatures of selection are a useful adjunct to trait mapping.

The region associated with the peak in F_ST _on bovine chromosome 2 contains several genes that have been associated with selective sweeps in humans. The genes *R3HDM1 *(R3H domain containing 1) and *ZRANB3 *(Zinc finger, RAN binding domain containing 3) are associated with these cattle SNP. The functions of these genes are inferred based on their sequence structures, and references to them point to the bioinformatics literature (e.g. Gene Ontology AmiGO  R3HDM1 GO:0004866 endoproteinase inhibitor checked 5 May 2008 [47]). For these particular SNP with high F_ST _values, most breeds are homozygous for the same allele, but the Hereford, the Santa Gertrudis and the Belmont Red have a moderate allele frequency of the alternative allele. The Hereford breed has been selected for efficiency and growth from its inception as a breed in the 18^th ^century and continues to be more efficient than other British beef breeds [48]. Polymorphisms in this region may therefore contribute to the differences seen in the Hereford. This region has long been known to be associated with positive selection in humans due to the lactase gene (*LCT*) and human adaptation to drinking milk in adulthood [9,10]. It is unlikely that cattle are selected for lactase persistence because breeding stock are invariably weaned. Recently, *R3HDM1 *[49] was also shown to be under positive selection in European humans, it is not divergent just through hitchhiking to *LCT*. These results in cattle provide a starting point for the kinds of trait that might be associated with the signal of selection in humans.

## Conclusion

Comparing the Australian and Bovine HapMap samples we found differences in the presumptive selective signatures when different breeds or SNP are used. This leads to the conclusion that a full description of signatures of selection in a species will require a large number of populations to be sampled, not just those that have the most divergent genotypes or phenotypes. In addition, SNP obtained from different genetic sources should also be used. A large amount of the variance in F_ST _across the genome is due to differences in SNP composition between studies, which suggests that evidence for selection in a region depends on the SNP that are included in a study. It also suggests that when the same genetic region is compared in different studies the same SNP should be used otherwise a large difference between selection signatures may be reported. These results also suggest that genome wide studies of selection are a useful adjunct to genome wide association studies. We found that useful signatures of selection can be obtained even when many of the sub-samples are not particularly divergent or cannot be clearly separated using a principal component analysis. If a sample used in a genome wide association study was genetically stratified, this stratification could be used to analyse gene frequency or F_ST _distributions in sections of the genome in different breeds or populations. Such an analysis may provide evidence for a selective signature or may even help to identify which populations are likely to contain the QTL even before functional nucleotide polymorphisms were identified. While many real QTL will not be accompanied by a signal of selection, such a signal will indicate a clear relationship between selection on some aspect of the phenotype and the effects of variation at a locus. The identification of the trait under selection will require animals of the appropriate breed or group measured for a wide range of traits. The use of evidence of presumptive selection on a genome wide scale means that panels of SNP will need to be several fold larger in cattle than currently available. Although several panels are now available that include 3.4–5.4 × 10^4 ^SNP in cattle (eg.  bovinesnp50, [14,50]), given the variation in F_ST _with distance between SNP, two to three times as many evenly spaced SNP will be needed to study signals of selection in all regions in detail.

## Authors' contributions

WB planned and performed the analyses, and drafted the manuscript, BH bled cattle, extracted DNA, RB bled cattle, extracted DNA, MT extracted DNA, LT, obtained cattle, led the bleeding of cattle. All authors reviewed the manuscript.

## Supplementary Material

Additional File 1 **Plot of experiment-wide statistics for allele frequency, average heterozygosity and F_ST_**. Two plots one above the other, the upper plot shows the average allele frequency plotted against the average heterozygosity for each SNP, the lower plot shows average heterozygosity plotted against F_ST _for each SNP. Click here for file

Additional File 2 **SNP with extreme F_ST _values in both the Bovine HapMap and Australian data sets**. A table consisting of six columns listing the dbSNP identifier, the chromosome, the position in bp in the Btau 4.0 assembly, the per locus F_ST _in the Australian and then the Bovine HapMap data, and the number of 1 Mb blocks per chromosome with loci with high or low F_ST_. Click here for file
