# Supplementary figures and images for: Genome wide signatures of positive selection: The comparison of independent samples and the identification of regions associated to traits
Source: BMC Genomics. 2009 Apr 24;10:178. doi: 10.1186/1471-2164-10-178 (PMC2681478; doi:10.1186/1471-2164-10-178)

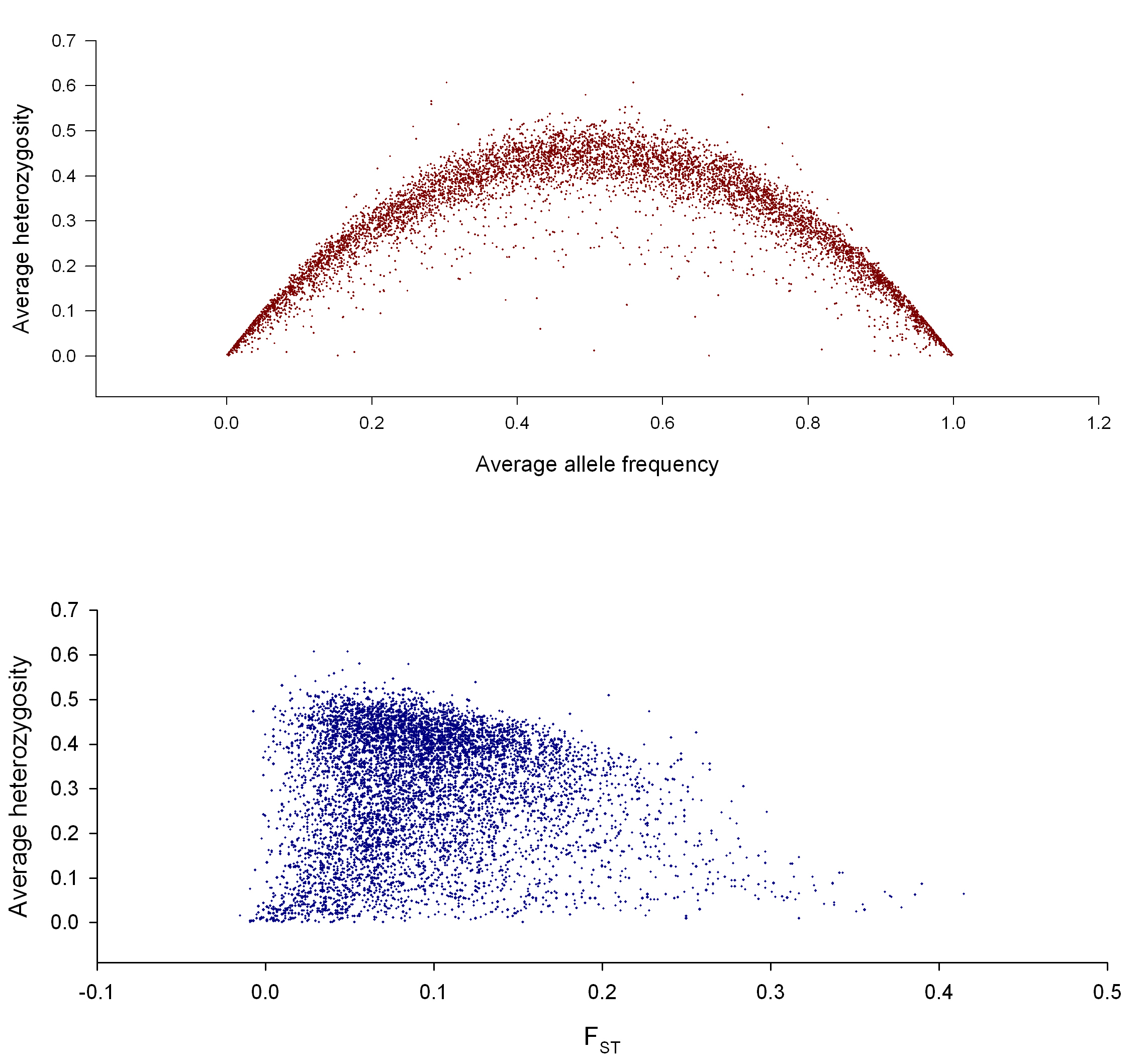

Supplement: Additional File 1 — Plot of experiment-wide statistics for allele frequency, average heterozygosity and FST. Two plots one above the other, the upper plot shows the average allele frequency plotted against the average heterozygosity for each SNP, the lower plot shows average heterozygosity plotted against FST for each SNP. [file 1471-2164-10-178-S1.jpeg]
