# Supplementary material for: Genome wide signatures of positive selection: The comparison of independent samples and the identification of regions associated to traits
Source: BMC Genomics. 2009 Apr 24;10:178. doi: 10.1186/1471-2164-10-178 (PMC2681478; doi:10.1186/1471-2164-10-178)
Supplement: Additional File 2 — SNP with extreme FST values in both the Bovine HapMap and Australian data sets. A table consisting of six columns listing the dbSNP identifier, the chromosome, the position in bp in the Btau 4.0 assembly, the per locus FST in the Australian and then the Bovine HapMap data, and the number of 1 Mb blocks per chromosome with loci with high or low FST. [file 1471-2164-10-178-S2.pdf]

Additional File 2.

Genome wide signatures of positive selection: The comparison of independent samples and the identification of regions associated to traits. BMC Genomics accepted October 2008. Barendse W, Harrison BE, Bunch RJ, Thomas MB, Turner LT.

SNP with extreme F_{ST} values in both the Bovine HapMap and Australian data sets.

**Locus**	**Chromosome**	**Position**	**F_{ST} this study**	**F_{ST} Bovine HapMap**	**1 Mb blocks per chromosome**
**High F_{ST} values**					
rs29017243	Chr1	94552620	0.260	0.324	
rs29010367	Chr1	100797886	0.277	0.364	
rs29010368	Chr1	100797954	0.279	0.360	
rs29010370	Chr1	100797998	0.263	0.367	
rs29025998	Chr1	117239110	0.232	0.337	3
rs29021613	Chr2	40579645	0.225	0.314	
rs29014403	Chr2	49761603	0.266	0.395	
rs29019351	Chr2	64740286	0.313	0.380	
rs29019352	Chr2	64740428	0.314	0.381	
rs29021800	Chr2	64792978	0.312	0.383	
rs29015270	Chr2	80019527	0.227	0.322	
rs29015267	Chr2	80019771	0.240	0.324	4
rs29014816	Chr3	13934385	0.233	0.334	
rs29011277	Chr3	88185033	0.237	0.310	
rs29024847	Chr3	114618417	0.327	0.416	3
rs29011076	Chr4	11913823	0.242	0.285	
rs29010315	Chr4	22462513	0.234	0.334	
rs29013771	Chr4	54568493	0.268	0.318	
rs29012382	Chr4	69495410	0.226	0.345	4
rs29017027	Chr5	16144756	0.293	0.378	
rs29025811	Chr5	51065596	0.235	0.329	
rs29016809	Chr5	51770120	0.254	0.366	
rs29016813	Chr5	51770448	0.247	0.365	
rs29015212	Chr5	81548929	0.297	0.296	
rs29021747	Chr5	115318910	0.262	0.302	
rs29015304	Chr5	125087141	0.229	0.374	5
rs29027233	Chr6	33967205	0.341	0.372	
rs29027232	Chr6	33967229	0.343	0.372	
rs29026121	Chr6	38600494	0.266	0.315	
rs29015041	Chr6	88160023	0.297	0.286	3
rs29024503	Chr7	25760800	0.264	0.311	
rs29022787	Chr7	51136954	0.225	0.368	3
rs29019365	Chr8	15244255	0.244	0.334	
rs29010774	Chr8	17016654	0.337	0.381	
rs29016073	Chr8	32446240	0.371	0.301	
rs29014626	Chr8	52301026	0.386	0.394	
rs29025168	Chr8	99635999	0.293	0.353	
rs29010530	Chr8	104885923	0.274	0.330	
rs29010533	Chr8	104888840	0.266	0.332	6

rs29026560	Chr9	47205531	0.259	0.296	
rs29023173	Chr9	59305310	0.249	0.329	
rs29011074	Chr9	69688197	0.234	0.293	3
ss46526369	Chr10	4751255	0.293	0.366	
rs29022059	Chr10	44329810	0.269	0.323	
rs29022060	Chr10	44329837	0.244	0.323	
rs29021046	Chr10	62484593	0.233	0.334	
rs29014988	Chr10	69149924	0.227	0.347	4
rs29021601	Chr11	39253966	0.258	0.330	
rs29019760	Chr11	64371895	0.278	0.400	
rs29013770	Chr11	78803108	0.286	0.307	
rs29026884	Chr11	86777748	0.234	0.287	
rs29026883	Chr11	86777914	0.263	0.328	4
rs29018847	Chr12	9713260	0.249	0.330	
rs29015935	Chr12	16810706	0.283	0.352	
rs29011179	Chr12	43406630	0.245	0.348	
rs29019295	Chr12	46635688	0.259	0.319	4
rs29020369	Chr13	10218293	0.332	0.288	
rs29020370	Chr13	10218647	0.317	0.340	
rs29019461	Chr13	10423704	0.355	0.302	
rs29016959	Chr13	22767093	0.372	0.384	
rs29012117	Chr13	28925713	0.298	0.347	
rs29014450	Chr13	36360017	0.281	0.302	5
rs29024079	Chr14	43126764	0.227	0.340	
rs29019586	Chr14	46902020	0.266	0.361	
rs29019588	Chr14	46902349	0.257	0.353	
rs29026018	Chr14	59924544	0.240	0.300	
rs29027601	Chr14	72632952	0.285	0.350	4
rs29019566	Chr15	18646517	0.250	0.321	
rs29017211	Chr15	25472681	0.337	0.404	2
rs29026036	Chr16	23320813	0.265	0.308	
rs29026034	Chr16	23320981	0.225	0.301	
rs29016037	Chr16	30575585	0.390	0.362	
rs29016039	Chr16	30575950	0.273	0.288	
rs29021963	Chr16	31068223	0.415	0.413	
rs29013788	Chr16	63283574	0.368	0.401	4
rs29012677	Chr17	36428918	0.227	0.327	1
rs29018173	Chr20	71369970	0.256	0.332	
rs29018172	Chr20	71370022	0.262	0.360	
rs29018171	Chr20	71370040	0.254	0.332	1
rs29022043	Chr21	25155413	0.255	0.320	
rs29024337	Chr21	28633622	0.276	0.330	
rs29020829	Chr22	29199086	0.326	0.311	
rs29024065	Chr22	40139571	0.325	0.289	2
rs29022423	Chr24	16734115	0.301	0.371	
rs29022422	Chr24	16734137	0.338	0.378	
rs29022420	Chr24	16734180	0.307	0.369	
rs29022419	Chr24	16734288	0.301	0.372	
rs29020891	Chr24	42951736	0.287	0.353	
rs29020890	Chr24	42951766	0.297	0.354	2
rs29009898	Chr26	38545913	0.251	0.305	1
rs29018070	Chr28	9812769	0.231	0.335	
rs29010304	Chr28	24504312	0.236	0.289	2

rs29022139	Chr29	17109628	0.378	0.362	
rs29022136	Chr29	17109723	0.356	0.399	1
**Low F_{ST} values**					
rs29012718	Chr1	34411661	-0.001	0.028	
rs29019865	Chr1	92434645	0.011	0.007	
rs29020075	Chr1	126527179	0.010	-0.007	
ss46526384	Chr1	128455404	0.003	-0.000	3
rs29016991	Chr2	30526182	0.001	0.004	
rs29025558	Chr2	54689711	0.007	0.002	2
rs29019239	Chr4	114174215	0.012	0.026	1
rs29018352	Chr5	93564418	0.011	0.036	1
rs29015661	Chr6	12676368	0.011	-0.000	
rs29024817	Chr6	13520547	0.012	0.018	
rs29014057	Chr6	97914357	0.002	0.013	3
rs29021149	Chr7	22240287	0.007	0.011	
rs29017155	Chr7	97248925	0.012	0.028	
rs29012040	Chr7	109947142	0.004	0.021	
rs29012041	Chr7	109947258	0.004	0.021	3
rs29011742	Chr9	10126654	-0.003	0.017	
rs29010261	Chr9	87665546	0.013	0.032	2
rs29020613	Chr10	71913488	0.012	-0.003	
rs29013291	Chr10	73992679	0.012	-0.003	2
rs29025295	Chr12	61844028	-0.009	-0.004	
rs29022322	Chr12	74942777	0.007	-0.016	2
rs29024875	Chr13	19907640	-0.002	0.000	
rs29021123	Chr13	72076285	0.012	0.036	2
rs29016325	Chr17	16965701	0.001	0.037	1
rs29018263	Chr18	5326992	0.005	0.028	
rs29022891	Chr18	64492276	-0.001	0.038	2
ss46526683	Chr19	17985815	0.007	0.003	1
rs29019870	Chr20	12956157	0.014	0.028	1
rs29010492	Chr23	30238489	0.012	0.027	1
rs29025657	Chr27	43902917	-0.007	0.000	1
rs29011567	Chr28	8736524	-0.001	0.037	1
rs29010145	Chr29	1813619	0.006	0.026	
rs29022138	Chr29	17109634	0.004	-0.006	1
rs29011290	ChrX	73056128	0.010	0.020	
rs29026155	ChrX	80352324	-0.009	0.033	2
